# Full predicted energy from nutrition and the effect on mortality and infectious complications in critically ill adults: a protocol for a systematic review and meta-analysis of parallel randomised controlled trials

**DOI:** 10.1186/s13643-015-0165-5

**Published:** 2015-12-12

**Authors:** Emma J. Ridley, Andrew R. Davies, Carol Hodgson, Adam Deane, Michael Bailey, D. James Cooper

**Affiliations:** Australian and New Zealand Intensive Care Research Centre, School of Public Health and Preventive Medicine, Monash University, Level 6, The Alfred Centre, 99 Commercial Road, Melbourne, VIC 3004 Australia; Nutrition Department, Alfred Health, Commercial Road, Melbourne, 3004 Australia; Intensive Care Unit, Royal Adelaide Hospital, Adelaide, SA Australia; Discipline of Acute Care Medicine, University of Adelaide, Adelaide, SA Australia; Department of Intensive Care Medicine, The Alfred, Commercial Road, Melbourne, 3004 Australia

**Keywords:** Enteral nutrition, Parenteral nutrition, Nutrition, Energy, Critically ill, Systematic review, Meta-analysis

## Abstract

**Background:**

Whilst nutrition is vital to survival in health, the precise role of nutrition during critical illness is controversial. More specifically, the exact amount of energy that is required during critical illness to optimally influence clinical outcomes remains unknown. The aim of this systematic literature review and meta-analysis is to evaluate the clinical effects of optimising nutrition to critically ill adult patients, such that the entire predicted amount of energy that the patient requires is delivered, on mortality and other important outcomes.

**Methods:**

A systematic literature review and meta-analysis will be conducted by searching for studies indexed in Medical Literature Analysis and Retrieval System Online (MEDLINE), Excerpta Medica Database (EMBASE), Cumulative Index to Nursing and Allied Health Literature (CINAHL) and the Cochrane Library. Searches will be restricted to English. Studies will be considered for inclusion if they are a parallel randomised controlled trial investigating a nutrition intervention in an adult critical care population, where one arm delivers ‘full predicted energy from nutrition’ (defined as provision of ≥80 % of the predicted energy required) and the other arm delivers energy less than 80 % of the predicted requirement. Two authors will independently perform title screening, full-text screening, data extraction and quality assessment for this review. The quality of individual studies will be assessed using the ‘Risk of Bias’ tool, and to assess the overall body of evidence, a ‘Summary of Findings’ table and the Grades of Recommendation, Assessment, Development and Evaluation system will be used, all recommended by the Cochrane Library. Pending the study heterogeneity that is determined, a fixed-effect meta-analysis with pre-defined subgroup analyses will be performed.

**Discussion:**

Currently, it is controversial whether optimal energy delivery is beneficial for outcomes in critically ill patients. This systematic review and meta-analysis will evaluate whether delivering optimal energy to critically ill adult patients improves outcomes when compared to delivery of lesser amounts.

**Systematic review registration:**

PROSPERO CRD42015027512

## Background

Nutrition is vital to survival in health. In critical illness, however, the role of nutrition is less defined. More specifically, the exact amount of energy that is required during critical illness to optimally influence clinical outcomes remains unknown. Prolonged provision of nutrition below a patient’s individual nutrition requirements (including under provision of energy, specifically) can result in malnutrition. Whist the prevalence of malnutrition in critically ill patients is generally poorly documented, poorly defined, and varies depending on the criteria used, reports indicate that worldwide prevalence in hospitalised patients is between 20 and 50 % internationally [1]. Malnutrition is thus likely to be commonplace in critically ill patients. In the acute hospitalised population, malnutrition has been associated with many undesirable clinical consequences such as reduced immune function, increased length of hospital stay, impaired wound healing, muscle wasting and ultimately increased health care costs [1]. Conversely, it is known that excessive nutrition can lead to over provision of energy and result in adverse patient effects including increased metabolic stress, hyperglycaemia and deranged liver function [2].

Despite the known consequences of significant under- or overfeeding in critically ill patients, there is considerable uncertainty regarding the ideal amount of energy to provide to optimise outcomes. One of the most significant issues in studies of critical illness nutrition is that delivery of the full (or even near-full) predicted energy amounts (where the full amount of energy a patient is predicted to require is administered) has been uncommon. This often leads to all patient groups in nutrition studies receiving less than their full predicted energy requirements [3–7]. This occurs in clinical practice too, with a large international multicentre observational study including 158 intensive care units (ICUs) and 2946 patients indicating that only 45 % of predicted energy was provided by standard enteral nutrition (EN) alone, probably due to delays in commencement, intestinal dysfunction, and withholding of EN for medical procedures [8–10]. Several small studies have suggested improved clinical outcomes when energy delivery approximates full predicted energy requirements from nutrition; however, this evidence has not been translated into clinical practice [11–13].

Given the lack of clear evidence to make recommendations regarding the optimum amount of energy to provide to critically ill patients, we sought to conduct a systematic review to aggregate and summarise the evidence from the trials in this field to inform future research and clinical practice.

## Objectives

The aim of this systematic literature review and meta-analysis is to assess the effect of delivery of full predicted energy from nutrition on mortality and other important clinical outcomes in critically ill adults. ‘Full predicted energy from nutrition’ is defined as provision of ≥80 % of the predicted energy determined by any method, and the comparator will be the delivery of energy less than 80 % of the predicted requirement determined by any method.

The clinical question posed in this review is ‘Does the delivery of full predicted energy from nutrition influence mortality or other important clinical outcomes in critically ill adults compared to the delivery of less than full predicted energy from nutrition?’

## Methods/design

A rigorous systematic review and meta-analysis of randomised controlled trials (RCTs) will be conducted using the methodology detailed in the *Cochrane Handbook for Systematic Reviews of Interventions* [14] and the Centre for Research and Dissemination (CRD)’s *Guidance for Undertaking Reviews in Health Care* [15].

### Population

We will include studies in critically ill adult patients (≥16 years) irrespective of admission diagnosis that receive enteral and/or parenteral nutrition (PN).

Study participants will be defined as ‘critically ill’ if they meet one of the following criteria (adapted from Simpson and Doig 2005 [16]):

1. The patients were recruited in an ICU.
2. The inclusion criteria specified in the study deemed that the patients would be required to be cared for in an ICU, i.e. invasive organ support.
3. The patients had an average ICU length of stay of greater than or equal to 2 days.

### Interventions and comparators

The intervention group (delivery of full predicted energy from nutrition) will comprise study arms which report that patients received a mean energy delivery of ≥80 % of estimated or measured energy requirements. Energy delivery must be provided by EN and/or PN but may also include some non-nutritional calorie sources such as propofol and dextrose. The alternate arm will be the control (comparator) group (mean energy delivery <80 % of full predicted energy requirements).

The metric of ≥80 % of estimated or measured energy requirements was chosen by the authors as it is above the international mean for energy delivery, it approximates energy requirements and observational evidence is emerging that receipt of ≥80 % of estimated energy requirements may improve clinical outcomes in certain patient groups [8, 17].

### Outcome measures

#### Primary

1. Hospital mortality
2. Hospital length of stay
3. Infectious complications

Infectious complications will be defined as any confirmed infectious event after randomisation, reported as the total number of events for each arm of the RCT, if an objective measure is described (i.e. positive blood culture).

#### Secondary

1. ICU and 90-day mortality
2. Duration of mechanical ventilation in survivors and non-survivors, measured in days
3. ICU and hospital length of stay in survivors and non-survivors, measured in days

### Inclusion criteria

Trials will be screened based on the following inclusion criteria:

1. Parallel group randomised controlled trials.
2. Mortality, hospital length of stay and/or infectious complications have been reported.
3. Patients were randomised into the study within 72 h of admission and nutrition therapy was commenced to both arms within 72 h of admission or appears to have been if it is not precisely reported.
4. Energy delivery from EN, PN or any combination is reported as a proportion of estimated requirements (by any method) or the required data to calculate this is provided.
5. One arm of the trial reports mean energy delivery of ≥80–120 % of the estimated or measured energy requirements, and the other reports mean energy delivery of <80 % of the estimated or measured energy requirements.
6. Conducted in adult (≥16 years) critically ill patients.
7. Both study arms received carbohydrate, lipid and protein as part of nutrition therapy.
8. The primary intervention in the research study was delivered as a component of nutrition therapy.

Abstracts alone, where the subsequent primary publication cannot be located, will be excluded.

We will not include cluster-randomised trials, non-randomised or quasi-randomised trials. We will also exclude cross-over trials because this methodology will not allow us to investigate the outcomes we have chosen.

### Search strategy

The current issues of the Cochrane Central Register of Controlled Trials (CENTRAL), Medical Literature Analysis and Retrieval System Online (MEDLINE) (Ovid SP, from 1948 to date), Excerpta Medica Database (EMBASE) (Ovid SP, from 1948 to date) and the Cumulative Index to Nursing and Allied Health Literature (CINAHL) (EBSCOhost, from 1948 to date) will be searched. Sensitivity-maximising strategies will be applied for each database as described in the Cochrane Handbook for Systematic Reviews of Interventions [14], and advice from a senior librarian with extensive knowledge in the area of medical systematic review will be sought. Publication restrictions for English language and studies containing adults and humans will be used pending the accuracy of the indexing for each search engine and at the advice of the senior librarian. Appendix demonstrates the MEDLINE search strategy which will be adopted for other search engines. Once included studies are identified, the reference lists, other systematic reviews on similar topics and clinical practice guidelines will be hand-searched to identify any other potential articles.

### Study selection and management of review processes

The EndNote reference manager software program (version X7.3, New York City: Thomas Reuters, 2011) and online systematic review management program, Covidence 2013 (www.covidence.org) will be used to coordinate the screening and data collection process. Covidence allows multiple authors to independently conduct the processes associated with a systematic review and then resolve any conflicts whilst tracking processes.

At each stage of study screening, selection for inclusion and exclusion criteria and data extraction processes, two authors will independently pilot suggested processes on 10 papers and then discuss to assess agreement, refine processes and ensure there is consistency in methodology. Once a final methodology has been agreed upon, it will be imputed into Covidence and used for the full set of articles as a final version at each stage.

### Study selection

Results of the searches described above will be merged in the reference manager software. Selection of relevant articles will be conducted in stages.

- Stage 1: Remove duplicates
  Using the reference manager software, one author will remove obvious duplicate articles from the initial search.

- Stage 2: Remove irrelevant articles
  Using the reference manager software one author will remove obviously irrelevant articles and those which are not RCTs (editorials, letters, abstracts, reviews, meta-analysis). This will be checked by the second author and over inclusion for screening will be preferred to exclusion at this stage.
  The final list after removal of duplicates and irrelevant articles will be uploaded into Covidence systematic review software for screening by two authors independently.

- Stage 3: Determine a final list of potentially relevant articles
  Two authors will then independently screen titles and abstracts using the Covidence software to determine a final list of potentially relevant studies for full-text review. After this screening process, the results will be compared and any conflicts resolved by discussion. Where eligibility cannot be determined using the abstract alone, the article will remain in consideration and the full text will be obtained. In the case of inability to reach consensus, a third review author will be consulted. Once a list of potentially relevant articles has been produced, the full text will be retrieved.

- Stage 4: Retrieve full-text reports for compliance of studies and determine eligibility criteria
  The same two authors will independently assess the full-text articles using a hierarchy of inclusion criteria previously outlined. The reasons for exclusion and any conflicts will be independently noted.

Conflicts will be resolved by discussion, with the two authors using the hierarchy of inclusion criteria to reassess the articles together in an attempt to obtain a consensus for inclusion or exclusion. In cases of inability to reach a consensus, a third review author will be asked to independently assess the article. A final list will be compiled of all eligible studies along with a list of excluded studies based on the review of the authors. The reference lists of the included studies and systematic reviews of similar topics (with and without meta-analysis) will also be checked to ensure there are no missing relevant articles. The final list of included and excluded studies will be discussed with the whole authorship group and any articles that may have been excluded but would have been expected to be included prior to assessment will be presented to ensure we have consensus and a ‘characteristics of excluded studies’ table will be developed. Any eligible studies will also be reviewed and compared at this stage to ensure there are no duplicate reports of studies.

### Data extraction

Study data extraction points will be developed based on the Cochrane Collaboration Study Selection and Data Extraction form [14]. These study characteristics will be pre-specified prior to data extraction and relate to patient and setting characteristics, study methodology, detailed data on nutrition therapy (including mode delivered, energy requirements (estimated or measured), energy delivered and duration of nutrition therapy) and detailed data on outcome variables such as how they were defined, how they were reported, the sample sizes in each group, missing data and any other relevant comments on each paper.

### Assessment of bias in included studies

Two review authors will independently assess the risk of bias in included articles using the Cochrane risk of bias tool, with a particular focus on sequence generation, allocation concealment, blinding, incomplete or selective reporting of outcome data and other sources of bias as recommended in the Cochrane Handbook for assessment of parallel trials [14]. To ensure consistency in assessment between authors, we will use the exact instructions provided by Cochrane for each domain, as set out in the Cochrane Handbook [14]. Where there is disagreement between author assessments, a conservative approach will be favoured, where article quality will be downgraded in the first instance. If consensus cannot be reached, a third author will be required to assess the article(s).

### Assessment of reporting biases

If 10 or more studies are identified, funnel plot, as recommended by Egger [18], will be created using the statistical software of The Cochrane Collaboration, Review Manager (RevMan) 5.3 [19]. We will conduct sensitivity analyses to explore the robustness of the meta-analysis in terms of conclusions related to the causes of funnel plot asymmetry.

### Data synthesis

Only available data will be synthesised; no missing date will be imputed. The included data will be quantitatively reviewed and combined by energy delivery for each specified primary and secondary outcome using RevMan 5.3 [19]. We will synthesise these data only in the absence of important clinical or statistical heterogeneity (see definition of important heterogeneity under ‘Assessment of statistical heterogeneity*’* below).

### Unit of analysis issues

We will include in our review only RCTs with a parallel-group design. The issue of repeated measures is not relevant for the outcomes under investigation.

Where different scales are used to measure the same outcome, we will present the treatment effect as the standardised mean difference (SMD) with 95 % confidence intervals (CIs). Measurements in different units will not be combined.

### Assessment of statistical heterogeneity

We will consider the *χ*^2^ statistic to test statistical heterogeneity between studies and will consider a *P* value ≤0.10 as indicating significant statistical heterogeneity; we will use the *I*^2^ statistic to assess the magnitude of heterogeneity [14]. We will consider *I*^2^ > 50 % to indicate problematic heterogeneity between studies and will carefully consider the value of any pooled analysis. If *I*^2^ is greater than 50 %, we will use a random-effects model analysis to determine the best estimate of the intervention effect; otherwise, a fixed-effect model of analysis will be used. If the two do not coincide, we will not consider the random-effects estimate as the actual intervention effect in the population under study. We will construct forest plots to summarise findings from the included studies.

### Statistical analysis and measures of treatment effect

The analysis will be undertaken using RevMan 5.3 software [19].

One of the primary outcomes (mortality) and one of the secondary outcomes (infectious outcomes) is binomial, whilst all other outcomes are continuous.

For binomial outcomes, we will present the treatment effect as an odds ratio (OR) with 95 % CIs.

For continuous outcomes, we will present the treatment effect as a mean difference (MD) or SMD with 95 % CIs. If variables are found to be non-normally distributed, appropriate statistical methods will be utilised for analysis where possible.

In addition to analysing the primary outcome variable (mortality) as a binomial variable, should sufficient data exist, we will also conduct time to event analysis for survival with results reported as hazard ratio (HR) with 95 % CIs in accordance with Tierney at al. and as specified in the Cochrane Handbook [14, 20]. Analysis using this data will be conducted using the generic inverse-variance method and the fixed and random effect analyses compared.

Subgroup analyses have been defined a priori. If obvious unexplained heterogeneity is observed (*I*^2^ > 50 %) between studies, we will consider other subgroup analysis and report these separately. Further, where studies of high quality and at low risk of bias exist, we will consider doing a separate analysis with these studies alone to further investigate any clinical effect. All analyses will be presented in the final paper. Where subgroup analyses are performed, the method described by Deeks and recommended by Cochrane will be used [14, 21].

### Subgroup analyses

A priori, and pending study numbers, we would like to conduct subgroup analysis of:

- Studies using only EN in the intervention group
- Studies using only PN in the intervention group
- Studies using EN and PN in the intervention group
- Trials investigating immunonutrition as the primary intervention
- Studies assessed as high quality and low risk of bias

These subgroups have been chosen as it is plausible that the mode of nutrition therapy may affect the specified outcomes differently. Immunonutrition has been pre-specified as the literature in this area is conflicting (and may include harm in the critically ill [22]) and so separate analysis is warranted.

### Summary of findings and quality of the body of evidence

We will present study findings in a standard ‘Summary of Findings’ (SOF) table, which will include the magnitude of effect, the numbers of participants and studies addressing each outcome and a grade for the overall quality of the body of evidence for each outcome. Space will be provided for comments.

We will use the principles of the Grades of Recommendation, Assessment, Development and Evaluation system [23] to assess the quality of the body of evidence associated with specific outcomes.

## Publication

The results of the meta-analysis will be published in a peer reviewed journal with all contributors listed as authors.

## Discussion

### Inform future studies

This systematic review and meta-analysis will inform the design of future nutrition studies investigating the relationship of energy dose in critical illness. We will also identify the gaps in the literature and trial design in relation to energy dose in nutrition research, which may assist in improving methodological quality of future studies. Nutrition is a universally provided standard of care to the critically ill and is inexpensive compared to other therapies, but the risks and benefits to patient outcomes are remarkably poorly understood.

### Expected benefits of this review

This will be the first published systematic review and meta-analysis to our knowledge that will investigate the effect of delivering full predicted energy from nutrition on clinical outcomes in critically ill adults, compared to delivering less than full predicted energy requirements. The literature available on this topic is conflicting and confusing for clinicians and could potentially lead to misleading conclusions being made regarding the role of nutrition in critical illness. This systematic review and meta-analysis will benefit clinicians by providing a summary of the available literature and provide further guidance.

## Appendix

**Table 1:** Sample search strategy for MEDLINE (other searches will be based on this)

Number	Searches
1	(((intensive or critica*) adj3 (care or unit* or illness*)) or ICU or (critical* adj ill) or (mechanical* adj4 ventilat*)).tw.
2	(artificial* adj2 (respirat* or ventilat*)).tw.
3	exp critical illness/ or exp critical care/ or exp intensive care/ or exp respiration, artificial/ or exp ventilation, mechanical/ or exp critical care nursing/ or exp intensive care units/
4	exp Multiple Organ Failure/ or exp Systemic Inflammatory Response Syndrome/
5	(multiple organ dysfunction* or multiple organ failure* or multi-organ failure* or Systemic Inflammatory Response or septic shock or sepsis syndrome*).tw.
6	Respiratory Distress Syndrome, Adult/
7	Respiratory Distress Syndrome*.tw.
8	1 or 2 or 3 or 4 or 5 or 6 or 7
9	((calorie* or energy) and (nutrient* or nutrition* or diet*)).tw.
10	(calori* or energy or underfe* or overfe* or hypercaloric or undernutrition or underprescription).tw.
11	exp intubation, gastrointestinal/
12	exp energy intake/ or exp caloric restriction/ or exp nutrition assessment/ or exp nutritional requirements/ or exp nutritional support/ or exp nutritional status/ or exp parenteral nutrition/ or energy metabolism/ or basal metabolism/ or nutrition therapy/ or exp enteral nutrition/
13	((parenteral* or intravenous*) adj2 (feed or feeding or feeds or fed or nutrition)).tw.
14	((enteral* or enteric or nasogastric) adj2 (feed or feeding or feeds or fed or nutrition)).tw.
15	(nutrition assessment* or nutrition* requirement* or nutrition* support* or nutrition* status or basal metabolism or nutrition* therap*).tw.
16	exp dietary supplements/
17	9 or 10 or 11 or 12 or 13 or 14 or 15 or 16
18	8 and 17
19	(randomized controlled trial or controlled clinical trial).pt. or (random* or trial or placebo).tw. or clinical trial*.pt.
20	18 and 19
21	exp animals/ not humans.sh.
22	(child* or infan* or pediatr* or paediatr* or neonat* or preterm or newborn* or NICU).mp.
23	20 not (21 or 22)
24	limit 23 to english language

## Abbreviations

CENTRAL: Cochrane Central Register of Controlled Trials
CI: confidence interval
CINAHL: Cumulative Index to Nursing and Allied Health Literature
CRD: Centre for Research and Dissemination
EN: enteral nutrition
HR: hazard ratio
ICU: intensive care unit
MD: mean difference
MEDLINE: Medical Literature Analysis and Retrieval System Online
OR: odds ratio
PN: parenteral nutrition
RCT: randomised controlled trial
RevMan: Review Manager
SMD: standardised mean difference
SOF: Summary of Findings
